# Retrovirus Integration into the Human Genome

**DOI:** 10.1371/journal.pbio.0020281

**Published:** 2004-08-17

When gene therapy was introduced nearly fifteen years ago, it was widely hailed as a panacea. Since many diseases have a genetic component, the hope was that gene therapy could replace compromised genes with healthy versions to treat everything from inherited disorders like cystic fibrosis to cancer and HIV. That great promise was quashed when a teenager suffering from a rare hereditary liver disorder died after participating in an experimental gene therapy trial in 1999: four days after being injected with millions of viruses engineered to deliver healthy genes to his liver, Jesse Gelsinger died. It seems the virus, derived from an adenovirus, targeted his immune cells rather than his liver cells, which triggered an immune response against the virus, resulting in massive organ failure.

In another case, two young boys who received gene therapy for the severe immunodeficiency disorder known as “bubble boy disease” developed leukemia-like symptoms 30 months after treatment. In this case, the viral vector inserted itself near a promoter region—a site that initiates gene transcription—of a proto-oncogene, a gene that can initiate cancer. Since viral vectors can integrate at various genomic locations, the safety and effectiveness of gene therapy ultimately depends on being able to predict a virus's particular bias. Comparing retroviral vectors derived from three viruses, including two common gene therapy vectors, Rick Mitchell et al. report 3,127 sites where these viruses typically integrate into the human genome. The different vectors, they found, show different target preferences.

Retroviruses use viral enzymes to copy their own genome, which is stored in an RNA transcript, into DNA. Now recognizable by the host's genome, the virus can integrate into one of the host's chromosomes. In this study, Mitchell et al. studied vectors derived from the human immunodeficiency virus (HIV), avian sarcoma-leukosis virus (ASLV), and murine leukemia virus (MLV). Introducing the viral vectors into human cells, the authors analyzed the gene expression profiles of the cells to determine where vectors integrate into human chromosomes and which, if any, genes they activate. Mitchell et al. then compared the integration sites with the transcription profiles.

Each retrovirus, they discovered, showed distinct preferences for genome integration. HIV vectors tend to integrate into sites of active transcription, favoring chromosomal regions rich in expressed genes. MLV vectors tend to integrate near transcription initiation sites, confirming the results of a previous study, with a weak bias toward active genes. In contrast, the authors report, the ASLV vector “does not favor integration near transcription sites, nor does it strongly favor active genes.”

Early efforts to understand how chromosomes may influence where viruses insinuate themselves into a chromosome focused on factors governing accessibility. Viruses are more likely to be integrated into chromosomal regions that are more accessible, which tend to be transcriptionally active sites. But since each of the three viruses studied here routinely targeted different sequences, the authors note, accessibility is probably just one factor. Specific chromosomal proteins, for example, might interact with the viral integration machinery and facilitate integration at nearby sites. Another possibility, the authors propose, is that DNA-binding proteins that bind to specific DNA sequences assist integration of one virus while impeding another.

This could explain why ASLV behaved as it did in the human cells studied here. The virus might have more refined integration preferences during normal infection of chicken cells, the authors note, but its integration machinery can't interact properly with human cells. The leukemia-like effects of the bubble boy gene therapy stemmed from integration of a mammalian retrovirus—the MLV vector—near an oncogene promoter region. Since ASLV tends to avoid both transcription initiation sites and active gene sites, it could be a more promising candidate for human gene therapy. With the draft chicken genome sequence now complete, researchers can investigate whether that proves true. But for now, Mitchell et al. make the case that scientists can gain more control over where viral vectors integrate into the human genome by selecting different retroviral integration systems. Only time will tell whether more control translates into safer gene therapy protocols.

